# 1900. Disseminated and Extrapulmonary Nontuberculous Mycobacteria (NTM): Disease Management, Adverse Treatment Reactions, and Outcomes

**DOI:** 10.1093/ofid/ofad500.1728

**Published:** 2023-11-27

**Authors:** Heather Wang, Mohammad Mahdee Sobhanie, Courtney Nichols

**Affiliations:** Rutgers New Jersey Medical School, Newark, New Jersey; The Ohio State University, Columbua, Ohio; OSU Wexner Medical Center, Columbus, Ohio

## Abstract

**Background:**

Guidelines for the management of pulmonary nontuberculous mycobacterial (NTM) infections are well defined; however, similar guidelines for the treatment of disseminated or extrapulmonary NTM infections are lacking. This study aimed to describe our institutional experience in the treatment of disseminated and extrapulmonary NTM infections, and to better characterize the disease course, management, and outcomes in this patient population.

**Methods:**

This single-center retrospective study was conducted at The Ohio State University Wexner Medical Center in patients who were diagnosed with disseminated or extrapulmonary NTM infection from November 1, 2011 to November 1, 2021. Only index cases were included in patients with a documented culture positive for an NTM, and patients with a positive pulmonary culture were only included if they had a documented extra-pulmonary site as well.

**Results:**

58 patients met inclusion criteria and 54 were started on antibiotic therapy. 41.4% (N=24) and 31.0% (N=18) were started on two and three antibiotics as initial therapy, respectively. 32 (59.3%) patients required changes to their initial regimen, of which 31.3% (N=10) were changed due to intolerance or adverse event. The mean time to onset of antibiotic intolerance or adverse drug events was 129.4 days (6-404 days). 67.2% (N=39) of patients had documented resolution of their infections, with 7 patients currently on antibiotic therapy at the end of the study period (38.9%).

The most common antibiotic classes used included macrolides (64.8%), carbapenems (35.2%), ethambutol (25.9%), and aminoglycosides (22.2%). The antibiotic classes with most frequent intolerances were linezolid (42%), aminoglycosides (31.6%) and clarithromycin (21.0%) at a mean duration of 39.3, 72.2, and 32 days, respectively.

**Figure:**
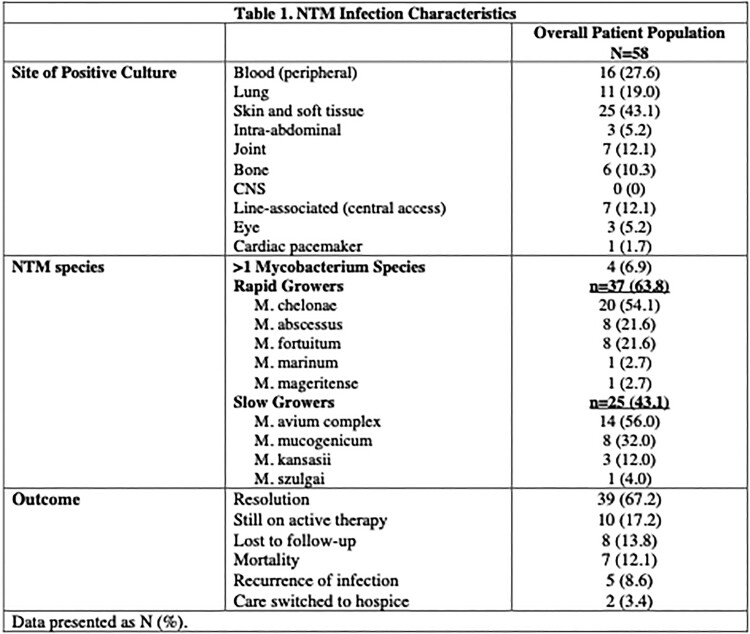

Table 1. NTM infection characteristics, including infection site, species breakdown, and disease outcome.

**Figure:**
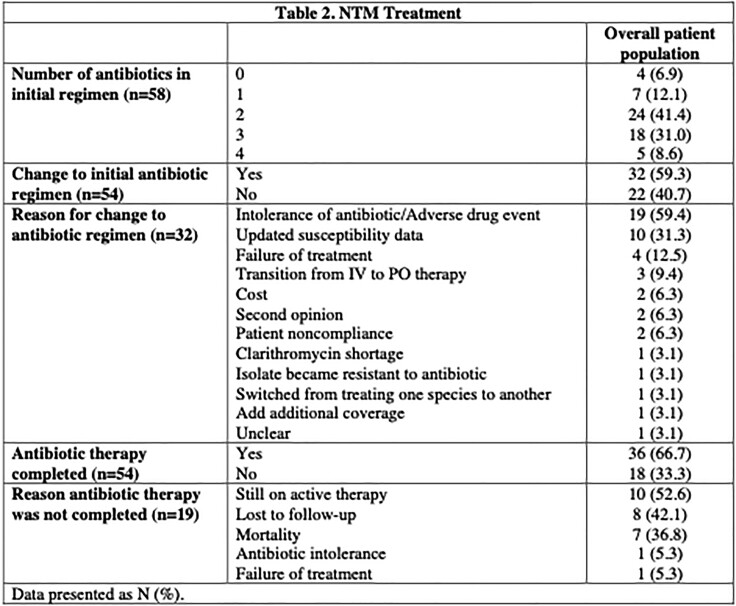

Table 2. NTM treatment regimen details and reasons for changing and/or not completing planned antibiotic regimen for all study patients.

**Figure:**
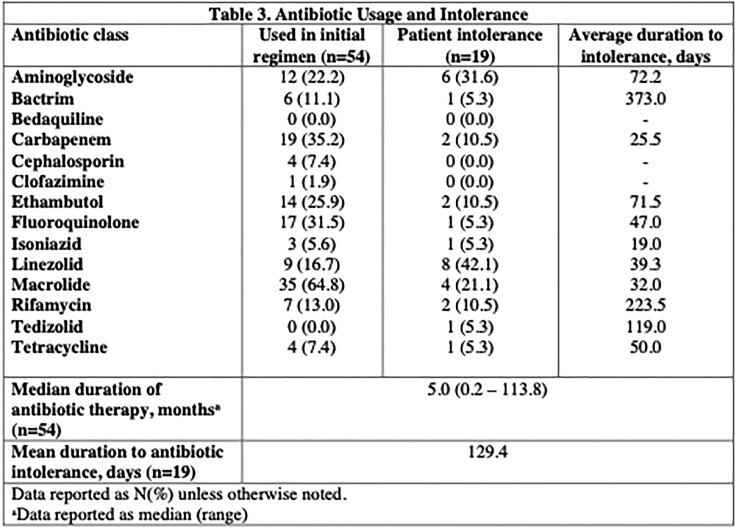

Table 3. Breakdown of antibiotic classes used in initial treatment regimens for dNTM-infected patients, along with antibiotics that caused patient intolerances. For each antibiotic class that caused intolerance, average time to develop intolerance listed.

**Conclusion:**

Treatment of disseminated and extrapulmonary NTM infections typically requires a complicated treatment course due to prolonged durations of multiple antibiotics, patient intolerances to antibiotic classes, and difficulty with determining appropriate end of therapy timing. Additional studies are needed to better outline the ideal antibiotic regimens and duration for disseminated and extrapulmonary NTM infections.

**Disclosures:**

**All Authors**: No reported disclosures

